# spiketools: a Python package for analyzing single-unit neural activity

**DOI:** 10.21105/joss.05268

**Published:** 2023-11-14

**Authors:** Thomas Donoghue, Sandra Maesta-Pereira, Claire Zhixian Han, Salman Ehtesham Qasim, Joshua Jacobs

**Affiliations:** 1 Department of Biomedical Engineering, Columbia University, New York, United States of America; 2 Department of Psychiatry, Icahn School of Medicine at Mount Sinai, New York, United States of America; 3 Department of Neurological Surgery, Columbia University, New York, United States of America

## Summary

A common method of collecting and analyzing neural activity is to implant electrodes that record the electrical activity of the brain, from which action potentials of individual neurons can be recorded (Buzsáki et al., 2012). After pre-processing to detect spike waveforms and cluster them into groups representing putative single neurons (Rey et al., 2015), this data can be used to investigate how neurons in the brain encode and process information. Analyzing single-unit activity requires dedicated analysis approaches, including representing spiking activity as spike times and/or binary spike trains, and analysis tools that allow for associating this activity to features of interest, for example the position of the subject in space or the properties of presented visual stimuli. To assist in this process, spiketools is a package designed to be used by neuroscientists for analyzing spiking activity.

spiketools is written in the Python programming language, built on existing tools in the scientific Python ecosystem, and developed using best-practice procedures. The module is accompanied by a documentation site that includes detailed tutorials for each of the modules, which are described below, as well as suggested workflows for combining them.

Modules in spiketools include:

- measures : measures and conversions that can be applied to spiking data
- objects : objects that can be used for managing spiking data
- spatial : space related functionality and measures
- stats : statistical measures for analyzing spiking data
- sim : simulations of spiking activity and related functionality
- plts : plotting functions for visualizing spiking data and related measures
- utils : additional utilities for working with spiking data

spiketools has the following required dependencies:

- numpy : used for managing and computing with arrays (Harris et al., 2020)
- scipy : used for some existing algorithms (Virtanen et al., 2020)
- pandas : used for managing heterogeneous data (McKinney, 2010)
- matplotlib : used for plotting (Hunter, 2007)

spiketools also has some optional dependencies that offer extra functionality:

- statsmodels : used for additional statistical measures (Seabold & Perktold, 2010)

## Statement of Need

spiketools is an open-source Python package for analyzing spiking neural data. Spiking neural activity is an idiosyncratic data stream with specific properties that requires specialized analysis tools including dedicated algorithms and statistical tools. Despite the popularity of this kind of data, there is currently a lack of openly available and maintained tools for this kind of data, especially within the Python ecosystem. spiketools therefore fills a niche, leveraging the power of the scientific Python ecosystem, while providing dedicated implementations for the specific requirements of spiking data.

Benefits of spiketools include that it follows modular organization, includes a test suite, follows a release cycle with versioned updates, and includes documentation and tutorials. spiketools is designed with a lightweight architecture in which functions take in arrays of spike times or spike trains, thus offering a flexible toolbox for custom analyses of spiking data. This approach also makes the tool flexible such that it can be integrated into existing codebases and workflows that use other tools. As part of the open-source Python ecosystem, spiketools also allows for sharing open-code that others can see and re-use. For example, spiketools has been demonstrated in an empirical project analyzing single-unit activity collected from human neuro-surgical patients, with openly available code showing all the analyses (Donoghue et al., 2023).

spiketools also offers a module for simulations, offering several methods for simulating spiking activity with specified parameters. Note that these simulations are designed to mimic the statistics of single unit spiking activity, but are not designed to replicate or reflect biophysical properties of neurons, and therefore should not be over-interpreted as biophysically realistic. Nevertheless, this simulation system allows for method testing, as new methods and implementations can be tested against synthetic data for which ground truth parameters are known.

## Related Projects

spiketools complements related tools that support other functionality in the ecosystem, including neo (Garcia et al., 2014), which supports loading and working with electrophysiological data, and spike interface (Buccino et al., 2020), which implements and supports spike-sorting related functionality. spiketools is designed with a lightweight architecture - whereby it manages data in common data types such as numpy arrays, without requiring any specific or idiosyncratic data formats. As such, this allows for integration with other related tools, for example, it could be used in combination with NeuroDSP (Cole et al., 2019), which provides functionality for analyzing neural time series, in order to examine relationships between spiking activity and the local field potential.

## Conclusion

The spiketools Python package offers functionality for analyzing single-unit activity that can be collected from human subjects and/or animal models, contributing to the ecosystem of scientific tools for analyzing neuroscience data.
